# Genomic and Transcriptomic Analysis of Nine Resurrection Plant Species: Conserved Regulation Versus Structural Paralog Capacity in Desiccation Tolerance

**DOI:** 10.3390/genes17101202

**Published:** 2026-09-29

**Authors:** Dimitar Kirkovski, Tsanko Gechev

**Affiliations:** 1 Center of Plant Systems Biology and Biotechnology, 4023 Plovdiv, Bulgaria; kirkovski@cpsbb.eu; 2 Department of Molecular Biology, University of Plovdiv, 4000 Plovdiv, Bulgaria

**Keywords:** comparative genomics, desiccation tolerance, LEA proteins, Mann–Whitney, orthogroups, resurrection plants, trend-conforming paralogs

## Abstract

**Background/Objectives**: Vegetative desiccation tolerance (VDT) has arisen independently in numerous angiosperm lineages, yet it remains unclear which of its molecular features are genuinely conserved beyond individual lineages. **Methods**: We compiled and compared available genome assemblies and *de novo* transcriptomes for the nine DT species and three desiccation-sensitive (DS) sister species. Conservatively regulated orthogroups were mapped onto eleven literature-established canonical desiccation-tolerance gene families, and species-level paralog representation was examined by principal component analysis and hierarchical clustering. An exact, one-sided Mann–Whitney test compared the number of trend-conforming paralogs per species between DT and DS groups across 32 canonical orthogroups. **Results**: Copy-number variation aligned more closely with taxonomic affiliation than with tolerance status itself. For instance, the expansion of the ELIP orthogroup (OG0000271) was particular to the Poaceae family instead of DT status. Of the 32 canonical orthogroups, three (dehydrin, a NAC-domain transcription factor, and a LEA hydroxyproline-rich glycoprotein) exhibited a nominally greater count of trend-conforming paralogs in DT compared to DS species using a genuinely exact one-sided Mann–Whitney test (uncorrected *p* < 0.05). None passed multiple-testing correction, a finding with a clear mathematical rationale: given the exact test’s combinatorial floor and a DS sample comprising only three species. **Conclusions**: The current analysis provides no statistically robust evidence that conserved desiccation-associated transcriptional responses are accompanied by systematic paralog enrichment in desiccation-tolerant species. We present an integrated transcriptomic-genomic analysis of paralog (“structural”) capacity, reflecting gene copies that follow each orthogroup’s dominant regulatory trend, whether identified from genome or *de novo* transcriptome data, in conservatively regulated gene families relevant to desiccation tolerance, together with a concrete, functionally diverse set of candidate genes for future targeted validation.

## 1. Introduction

Desiccation tolerance, the capacity to lose almost all cellular water and to resume normal physiological function upon rehydration, is widespread among seeds but exceptionally rare in the vegetative tissues of flowering plants [1]. Whereas most angiosperms die upon even mild dehydration, a small group of vascular plants, the “resurrection plants,” can survive dehydration to an air-dry state and recover fully within hours to days of rewatering [2,3]. Vegetative desiccation tolerance (VDT) has been described in only a few hundred angiosperm species, distributed across numerous independent phylogenetic lineages and representing well under 1% of flowering plant diversity [4]. This mixture of rarity and repeated, independent origin makes VDT a compelling system for studying convergent evolution: the same extreme phenotype has arisen many times, in distantly related taxa, under similar selective pressures.

The molecular mechanisms underlying VDT are complex and multi-layered. During dehydration, resurrection plants activate coordinated programs that protect cellular macromolecules and membranes: accumulation of protective proteins such as dehydrins and Late Embryogenesis Abundant (LEA) proteins, synthesis of osmoprotectants, induction of antioxidant systems that counter reactive oxygen species, remodeling of membrane lipids, and controlled dismantling of the photosynthetic apparatus [5]. A consistent genomic feature observed across separate lineages is the tandem amplification of Early Light-Induced Protein (ELIP) genes, which serves as a hallmark of convergent adaptation [6]. Another recurring pattern is the co-option of seed-specific protection mechanisms, such as the LEA/oleosin system, into vegetative tissues during drought. However, in *Xerophyta humilis*, VDT does not appear to have evolved simply through the reactivation of the canonical seed LAFL regulatory network [7,8].

Advances in sequencing has produced genomes for various VDT models. Among the first were *Dorcoceras hygrometricum* (also known as *Boea hygrometrica*) [9] and the diploid grass *Oropetium thomaeum*, later assembled to chromosome scale [10,11]. A high-quality genome of *Lindernia brevidens*, compared with its desiccation-sensitive relative *Lindernia subracemosa*, revealed that VDT can evolve through gene duplication and regulatory network rewiring [8]. The genome of *Xerophyta schlechteri* (published as *Xerophyta viscosa*; see Section 2) and that of *Sprobolus stapfianus* were released as part of comparative studies of tolerant and sensitive grasses [12,13], the genome of *Eragrostis nindensis* was published separately, alongside its desiccation-sensitive sister *Eragrostis tef* [14], and a high-quality genome of *Haberlea rhodopensis* was recently reported [15].

Most comparative genomic work to date has focused on single species, on paired tolerant-sensitive comparisons within a single lineage, or, as in a recent three-species comparison within the Chloridoideae grasses, on convergent evolution within a single clade [16]. What remains lacking is a broad comparative analysis integrating genomic and transcriptomic evidence across many VDT species spanning both major angiosperm clades: monocots and eudicots. We addressed this gap by examining, from a genomic perspective, the orthogroups conservatively regulated under desiccation in the nine DT species (Supplementary Methods S1) to distinguish molecular mechanisms actually conserved across distinct VDT origins from those unique to specific lineages.

We compiled and integrated available reference genomic and *de novo* transcriptomic resources for the studied species, addressing high data heterogeneity in assembly scale, contiguity, and predicted gene models. From the conservatively regulated orthogroups under desiccation, we identified those where tolerant species consistently harbor more paralogs following the dominant regulatory trend compared to their sensitive sister species. We then analyzed this subset of orthogroups using principal component analysis and hierarchical clustering. Finally, we identified whether literature-established canonical desiccation-tolerance orthogroups are represented within this subset and statistically tested whether tolerant species maintain a higher number of trend-conforming paralogs in these canonical groups than their sensitive counterparts, while discussing the results within the statistical constraints of our three-species sensitive control group.

## 2. Materials and Methods

### 2.1. Taxon Sampling

The study covers 20 angiosperm species: nine desiccation-tolerant (DT) species—the eudicots *Craterostigma plantagineum* (Cr), *D. hygrometricum* (Bh), *H. rhodopensis* (Hrh), and *L. brevidens* (Lb), and the monocots *E. nindensis* (En), *O. thomaeum* (Oth), *Sporobolus stapfianus* (Sst), *X. humilis* (Xh), and *X. schlechteri* (Xs); three desiccation-sensitive (DS) species forming phylogenetically matched sister pairs with three of the DT species—*L. subracemosa* (Ls, sister to Lb), *E. tef* (Etf, sister to En), and *Sporobolus pyramidalis* (Spr, sister to Sst); and eight species providing broader phylogenetic context—the models *Arabidopsis thaliana* (Ath) and *Oryza sativa* (Os), the basal angiosperm *Amborella trichopoda* (Amb), and five mesophytic species (*Brachypodium distachyon*, *Callicarpa americana*, *Lactuca sativa*, *Setaria italica*, *Trifolium pratense*), included to better resolve orthogroup boundaries. For *X. schlechteri*, we used the genome published by Costa [13] under the name *X. viscosa*; a subsequent taxonomic revision established that the sequenced accession corresponds to *X. schlechteri* (syn. *X. viscosa sensu* [17], a nomenclature we adopt throughout.

### 2.2. Genomic and Transcriptomic Resources and Assembly Quality

Publicly available genome assemblies and primary gene annotations were utilized for six DT species (Bh, En, Hrh, Oth, Sst, Xs) and two DS species (Etf, Spr) (Table 1). For the remaining four species—the DT species *Cr. plantagineum*, *L. brevidens*, and *X. humilis*, and the DS species *L. subracemosa*—no high-quality genome annotations were available at the time of analysis. Consequently, sequence identification and expression profiling for these four species relied entirely on *de novo* assembled transcriptomes. Transcriptomes for *C. plantagineum*, *L. brevidens*, and *L. subracemosa* were assembled *de novo* as detailed in Supplementary Methods S1, whereas the *X. humilis* transcriptome was retrieved from Lyall [18]. Raw data processing, assembly validation, differential expression analyses, and regulatory trend evaluations are fully described in Supplementary Methods S1. All reported gene and protein counts were derived from their respective primary sources or assembly annotations (Table 1). For the nine DT species, RNA-seq sampling spanned between three and six relative water content (RWC) time points per species, covering both the dehydration (RWC > 40%) and desiccation (RWC < 40%) stages defined in Section 2.4 (Supplementary Table S1). The NCBI SRA BioProject accessions for these RNA-seq datasets are listed in Supplementary Table S2.

### 2.3. Species-Tree Reconstruction

Orthogroups across all 20 species were delineated using OrthoFinder [19]. The species tree (Figure 1) was inferred by OrthoFinder from the resulting orthologous clusters and rooted on the basal angiosperm *A. trichopoda*. Visualization was performed in iTOL [20]. To assess statistical support for this topology, we independently reconstructed a maximum-likelihood phylogeny from a concatenated supermatrix of single-copy orthologs: per-orthogroup protein alignments were generated with MAFFT v7 [21], concatenated into a single supermatrix with AMAS 1.0 [22], and analyzed in IQ-TREE 2 v.2.2.0.7 [23,24] under ModelFinder-selected substitution models with 1000 ultrafast bootstrap replicates [25]. Branch support values for all major splits are reported in Supplementary Table S3.

### 2.4. Differential Expression and Identification of Conservatively Regulated Trends

Differential expression (DE) relative to the well-hydrated control was determined for each gene across individual stress samples, which were subsequently grouped into two physiological stages based on relative water content (RWC): dehydration (RWC > 40%) and desiccation (RWC < 40%); RWC was measured on a per-species, per-sample basis and used only to assign that species’ own samples to a stress stage, not to calibrate or compare expression directly across species. Genes were classified at *p*_adj_ <0.05 as upregulated (U; log_2_FC > 1), downregulated (D; log_2_FC < −1), or unchanged (N; −1 < log_2_FC < 1). To capture expression dynamics across species with multiple time points within the same stress stage (e.g., early vs. late dehydration), two parallel datasets were constructed using the minimum (“min”) and maximum (“max”) log_2_FC values per stage. From these, two-letter regulatory trends (XY) were defined, where X and Y represent the expression response under dehydration and desiccation, respectively. Up-oriented (UU, NU, UN, DU) and down-oriented (DD, DN, ND, UD) trends were evaluated, whereas non-differentially expressed genes (NN trend) were excluded. Orthogroups were delineated using OrthoFinder. For each orthogroup, the dominant trend was identified as the mode of individual gene trends, and a conservation score (Score) was calculated as the mean percentage of genes across the nine DT species conforming to this dominant trend. The 90th percentile corresponded to Score ≥ 35.19% (encompassing 2010 orthogroups; Supplementary Table S4). To confirm that this set reflects genuine cross-species regulatory conservation rather than random noise, we performed a species-level permutation test (1000 iterations) in which trend labels were randomly reshuffled within each species independently, preserving each species’ own trend-label frequencies while breaking inter-species coordination. The observed count of 2010 conservative orthogroups substantially exceeded the null expectation (mean = 956.9, SD = 23.4, across 1000 permutations; empirical *p* < 0.001), confirming that the identified conservative set reflects a genuine biological signal rather than a stochastic artifact of the 90th-percentile cut-off. Detailed trend construction and computational workflows are provided in Supplementary Methods S1.

### 2.5. Functional Annotation of Orthogroups at Gene Family Level

Functional annotation of orthogroups was systematically conducted at the gene family level using *A. thaliana* genes contained within each orthogroup as annotation anchors. Because *A. thaliana* is a desiccation-sensitive model, using it for functional assignment in resurrection species presents inherent limitations at the individual gene level; thus, functional inference was intentionally restricted to the broader gene family level. Gene family identifiers were programmatically retrieved by querying *A. thaliana* gene IDs against the family endpoint of the Ensembl Plants REST API v 15.12 [26], as described in Supplementary Methods S1. Here, “gene family” refers to the Ensembl Compara family classification returned for each *A. thaliana* gene by this endpoint, rather than a Gene Ontology-based grouping.

### 2.6. Literature Survey and Definition of Canonical Desiccation-Tolerance Gene Families

The canonical gene families summarized in Table 2 were compiled through a curated literature survey of the primary genome publications for the study species (Table 1), supplemented by key synthesis literature on plant systemic stress responses and vegetative desiccation tolerance. Gene families consistently recognized as core components of the drought and desiccation response were extracted to establish this benchmark set.

### 2.7. Mapping Conservatively Regulated Orthogroups to Canonical Families; PCA and Hierarchical Clustering

Conservatively regulated orthogroups were mapped onto canonical gene families using the functional annotations described above. To analyze representation patterns, we filtered for orthogroups exhibiting a higher gene copy number in the DT species relative to its sensitive counterpart across all three sister pairs (Lb/Ls, En/Etf, and Sst/Spr). Principal component analysis (PCA; Figure 2) was performed on the orthogroup-by-species copy-number matrix using the “prcomp” function in R, with variables mean-centered and scaled to unit variance. Hierarchical clustering and heatmap visualization (Figure 3) were generated using the pheatmap package [27], applying row-wise z-score standardization (scale = “row”) with default Euclidean distance and complete-linkage clustering.

### 2.8. Statistical Analysis of Trend-Conforming Paralog Dosage in Canonical Orthogroups: Mann–Whitney Test

For each orthogroup, the dominant regulatory trend established across the nine DT species (Section 2.4) served as the reference model, and genes conforming to this trend across all analyzed species were designated as trend-conforming paralogs. To test the hypothesis that DT species carry a significantly higher dosage of trend-conforming paralogs than DS species (“DT” > “DS”), species-level paralog counts were compared between DT (*n* = 9; Bh, Cr, En, Hrh, Lb, Oth, Sst, Xh, Xs) and DS (*n* = 3; Etf, Ls, Spr) species using an exact, one-sided Mann–Whitney *U* test (Wilcoxon rank-sum test). The test was applied to 36 orthogroups in total: 32 preselected canonical orthogroups constituting the primary analysis (Table 3; derived from the intersection of Section 2.6 and Section 2.7), and four additional canonical orthogroups from the same literature-established gene families (Table 2) that did not meet the conservative-regulation criterion of Section 2.7 but were tested alongside the primary 32 using the same procedure (Section 4.3.3). Because the primary 32 orthogroups were themselves selected in Section 2.7 on the basis of higher gene copy number in DT relative to DS species, this comparison is selection-conditioned and is interpreted as an exploratory, hypothesis-generating analysis rather than an independent statistical test of DT-associated enrichment. For comparative purposes, the same test was also evaluated genome-wide across 21,517 orthogroups. Raw *p*-values were adjusted for multiple testing using the Benjamini–Hochberg false discovery rate (FDR) procedure, applied independently to the canonical set (36 tests total) and the genome-wide set, using the p.adjust() function in R [28]. For conciseness, Table 3 reports the ten primary canonical orthogroups with the lowest adjusted *p*-values; complete results for all 32 primary canonical orthogroups are provided in Supplementary Table S5. Results and detailed biological interpretations are presented in Section 3.5 and Section 4.3.3.

### 2.9. Software and Versions

A comprehensive list of computational tools used for raw data download, quality control, *de novo* transcriptome assembly and annotation, orthogroup delineation, transcript quantification, and differential expression analysis is provided in Supplementary Methods S1. All statistical analyses and data visualizations were executed in R (v.4.5.2) [28]. Data wrangling and processing relied on the tidyverse suite (v.2.0.0) [29], including dplyr (v.1.1.4) [30] and purrr (v.1.1.1) [31]. Multivariate analyses and visualizations were generated using ggplot2 (v.4.0.2) [32], ggfortify (v.0.4.19) [33], pheatmap (v.1.0.13) [27], and iTOL [20]. Principal component analysis and the Mann–Whitney U test were performed using the “prcomp” and “wilcox.test” functions (R stats package v.4.5.2), with multiple testing corrections applied via “p.adjust”.

## 3. Results

### 3.1. Phylogenetic Relationships and Genomic Resources

The species tree (Figure 1), reconstructed from orthologous clusters across the 20 angiosperms, clearly separates the two clades under study, eudicots and monocots, and reproduces the three phylogenetically matched tolerant/sensitive pairs: *L. brevidens*/*L. subracemosa*, *E. nindensis*/*E. tef* and *S. stapfianus*/*S. pyramidalis*. Within the eudicot clade, *H. rhodopensis* and *D. hygrometricum* form a sister pair, while *Cr. plantagineum* is placed as sister to the Lindernia pair, although this specific grouping receives only moderate support (63% ultrafast bootstrap) in an independent maximum-likelihood reanalysis, which instead recovers *Cr. plantagineum* and *L. subracemosa* as the more closely related pair (Section 2.3; Supplementary Table S3), plausibly reflecting either reduced phylogenetic signal, since all three taxa in this clade (*Cr. plantagineum*, *L. brevidens*, and *L. subracemosa*) are represented by *de novo* assembled transcriptomes rather than genome annotations (Section 4.1), or a genuinely short, difficult-to-resolve divergence interval between these lineages. Among the monocots, *X. schlechteri* and *X. humilis* remain phylogenetically distinct from the other DT species, while *O. thomaeum* is nested among the Chloridoideae grasses [34] together with the En/Etf and Sst/Spr pairs.

**Figure 1:**
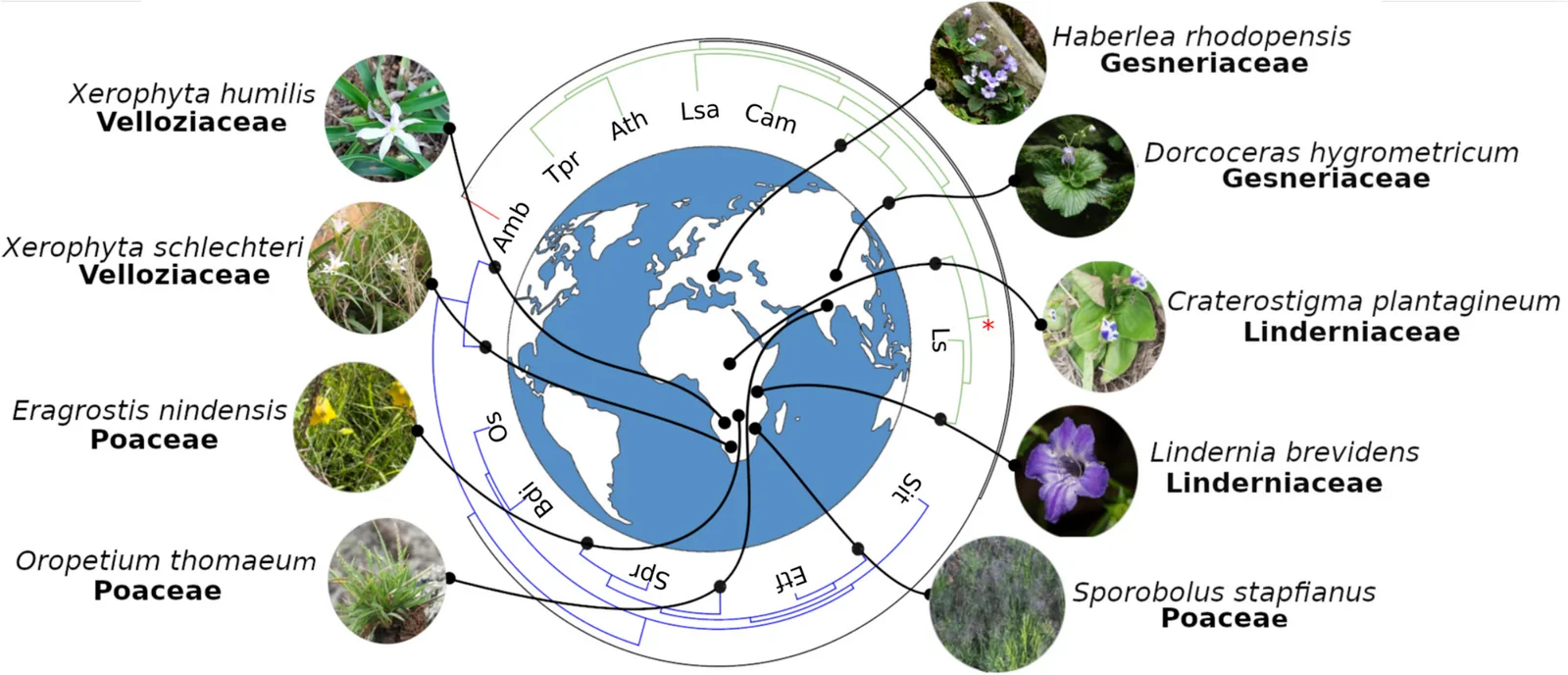
Global distribution and phylogenetic relationships of the studied angiosperms. Circular species tree reconstructed with OrthoFinder, showing the phylogenetic positions and geographic provenance of the 20 analyzed species. The eudicot clade is highlighted in green, the monocot clade in blue, and the basal angiosperm outgroup (*A. trichopoda*, Amb) in red. Connecting lines map each resurrection species to its location on the phylogeny and its primary native geographic distribution on the central map. Representative photographs depict the vegetative morphology of the desiccation-tolerant species. Branch support values (independently assessed by IQ-TREE 2 ultrafast bootstrap analysis of a concatenated single-copy-ortholog supermatrix; Section 2.3) are reported in Supplementary Table S3 rather than displayed on the tree itself. The asterisk (*) marks a branch with low bootstrap support (63%), corresponding to the placement of *Cr. plantagineum*.

Table 1 summarises the available genomic resources for the nine DT and three DS species and shows substantial heterogeneity in data quality. Assemblies with relatively high contiguity (scaffold N50 > 19 Mb) are available for *O. thomaeum*, *E. tef*, *S. pyramidalis* and *S. stapfianus*, whereas in *D. hygrometricum*, *H. rhodopensis* and *X. schlechteri* scaffold N50 is considerably lower (~114 kb, ~2.9 Mb and ~1.7 Mb, respectively) despite a higher contig N50 in Hrh and Xs. Of the nine DT species, *X. humilis* has no published genome sequence, while *Cr. plantagineum* and *L. brevidens* have genome sequence assemblies deposited in NCBI [8,35] but lack a reliable gene annotation, as does the DS species *L. subracemosa*; for all four, the analysis relies entirely on *de novo* assembled transcriptomes (Section 2.2). The number of predicted genes ranges from 21,055 (Lb, transcriptome) to 116,174 (En, tetraploid genome); for these four transcriptome-only species, gene numbers are 42,483 (Cr), 21,055 (Lb), and 74,881 (Xh), values comparable to the genome-sequenced species but likely underestimating the true gene repertoire (see Section 4.1). Transcription-factor counts are available for all genome-sequenced species, including *H. rhodopensis* (2507 TFs), and are missing only for the four transcriptome-only species. Ploidy in the sample is heterogeneous—from diploid (Bh, Lb, Ls, Hrh, Oth) to tetraploid (Etf, En, Xh, Sst) and octoploid (Cr, Xs) genomes—a combination of data type (genome versus transcriptome) and ploidy that represents a major source of heterogeneity when interpreting the paralog-count results (see Section 3.5 and Section 4.1).

**Table 1:** Summary of assembly statistics, gene model annotations, and chlorophyll-retention strategies for the analyzed DT and DS species.

Reference	TF	Genes	Scaffold N50	Contig N50	Genome Size	Chl	Ploidy	Species
			(Mb)	(Mb)	(GB)			
								Eudicots
[9]	1342	47,778	0.11	0.01	1.52	HDT	2n	*D. hygrometricum*
[35]	-	42,483 ^T^	-	-	1.70 ^U^	HDT	8n	*Cr. plantagineum*
[8]	-	21,055 ^T^	-	-	0.27 ^U^	HDT	2n	*L. brevidens*
[8]	-	72,851 ^T^	-	-	0.25 ^U^	-	2n	*L. subracemosa*
[15]	2507	44,306	2.92	1.10	1.27	HDT	2n	*H. rhodopensis*
								Monocots
[36]	3411	68,251	27.14	1.42	0.58	-	4n	*E. tef*
[14]	4345	116,174	0.52	0.52	0.99	PDT	4n	*E. nindensis*
[10]	1618	28,904	26.81	1.86	0.24	HDT	2n	*O. thomaeum*
[12]	2862	51,207	21.59	0.16	1.06	-	6n	*S. pyramidalis*
[12]	2691	52,808	19.42	0.01	1.08	HDT	4n	*S. stapfianus*
[13]	1905	25,425	1.67	1.11	0.300	PDT	8n	*X. schlechteri*
[18]	-	74,881 ^T^	-	-	*-*	PDT	4n	*X. humilis*

TF, transcription factor. Chl strategy: HDT, homoiochlorophyllous; PDT, poikilochlorophyllous; -, desiccation-sensitive. ^T^ Predicted gene/transcript counts derived from *de novo* assembled transcriptomes. ^U^ Genome sequence assembly is publicly available in NCBI, but lacks a reliable gene annotation (GFF3/GTF); gene models were therefore derived from *de novo* transcriptomes (see Section 2.2). No genome assembly is available for *X. humilis*.

### 3.2. Identification of Conservatively Regulated Orthogroups

The foundational dataset for this study comprises a set of conservatively regulated orthogroups (Supplementary Table S4), identified using the differential expression and orthology pipelines described in Section 2.4 and Supplementary Methods S1. Briefly, among orthogroups shared by all nine DT species, a core subset exhibits a conserved direction of regulation under dehydration. This conservatively regulated set serves as the baseline framework for the genomic analyses presented in Section 3.3, Section 3.4 and Section 3.5.

### 3.3. Representation Patterns and Clustering of Conservatively Regulated Orthogroups

To evaluate whether transcriptional conservation under dehydration aligns with gene family expansion, we examined a selected set of conservatively regulated orthogroups showing higher paralog representation across six functional categories: photoprotection and pigment control, LEA and dehydrin proteins, lipid and carbohydrate dynamics, the antioxidant system, autophagy and cellular recycling, and molecular chaperones (Supplementary Table S6).

Comparative evaluation reveals that paralog expansion does not uniformly correlate with desiccation tolerance (DT) status. For instance, in OG0000271 (chlorophyll a/b-binding proteins; ELIP1/ELIP2), the DT grasses *E. nindensis* (En, 28 copies) and *S. stapfianus* (Sst, 28 copies) exhibit marked expansions; however, the DS sister species *S. pyramidalis* (Spr) displays a nearly equivalent count (24 copies), whereas the eudicot DT/DS pair *L. brevidens* (Lb, 6 copies) and *L. subracemosa* (Ls, 5 copies) remains substantially lower (Supplementary Table S6). This pattern indicates that copy-number variance in this orthogroup primarily tracks lineage-specific evolutionary history (grasses vs. eudicots) rather than DT status per se.

Similarly, for OG0001470 (GSR/GSRL), the highest paralog counts occur exclusively within the genus Xerophyta (Xs = 20, Xh = 9), irrespective of DT/DS representation in other clades, highlighting a clade-specific lineage expansion. In the LEA/dehydrin category and the antioxidant system (e.g., class-zeta glutathione S-transferases: En = 12, Sst = 15, Spr = 10), a broad trend toward higher gene dosage is observed across Poaceae regardless of tolerance. Conversely, differences among species are more constrained among molecular chaperones, with the notable exception of HSP20-like chaperones (OG0003033), where Ls (DS) unexpectedly exhibits the highest copy number (10 copies) compared to all other analyzed species (1–4 copies).

Principal component analysis (PCA) of the six sister-pair species based on orthogroup copy number (Figure 2) accounts for 88.11% of the total variance (“PC1” = 74.65%, “PC2” = 13.46%). Separation along PC1 is primarily driven by *E. nindensis* at the extreme positive end, with *L. subracemosa* occupying the opposite negative pole, while *S. stapfianus* and *S. pyramidalis* cluster closely at lower PC2 values. Crucially, DT and DS species do not form distinct clusters along PC1. Lb (DT) and Ls (DS) cluster in close proximity, as do Sst (DT) and Spr (DS), indicating that copy-number profiles are more closely grouped by taxonomic lineage than by DT/DS status in this dataset; we note that PCA and hierarchical clustering are descriptive ordination methods here and do not themselves formally partition variance between phylogeny and tolerance status.

**Figure 2:**
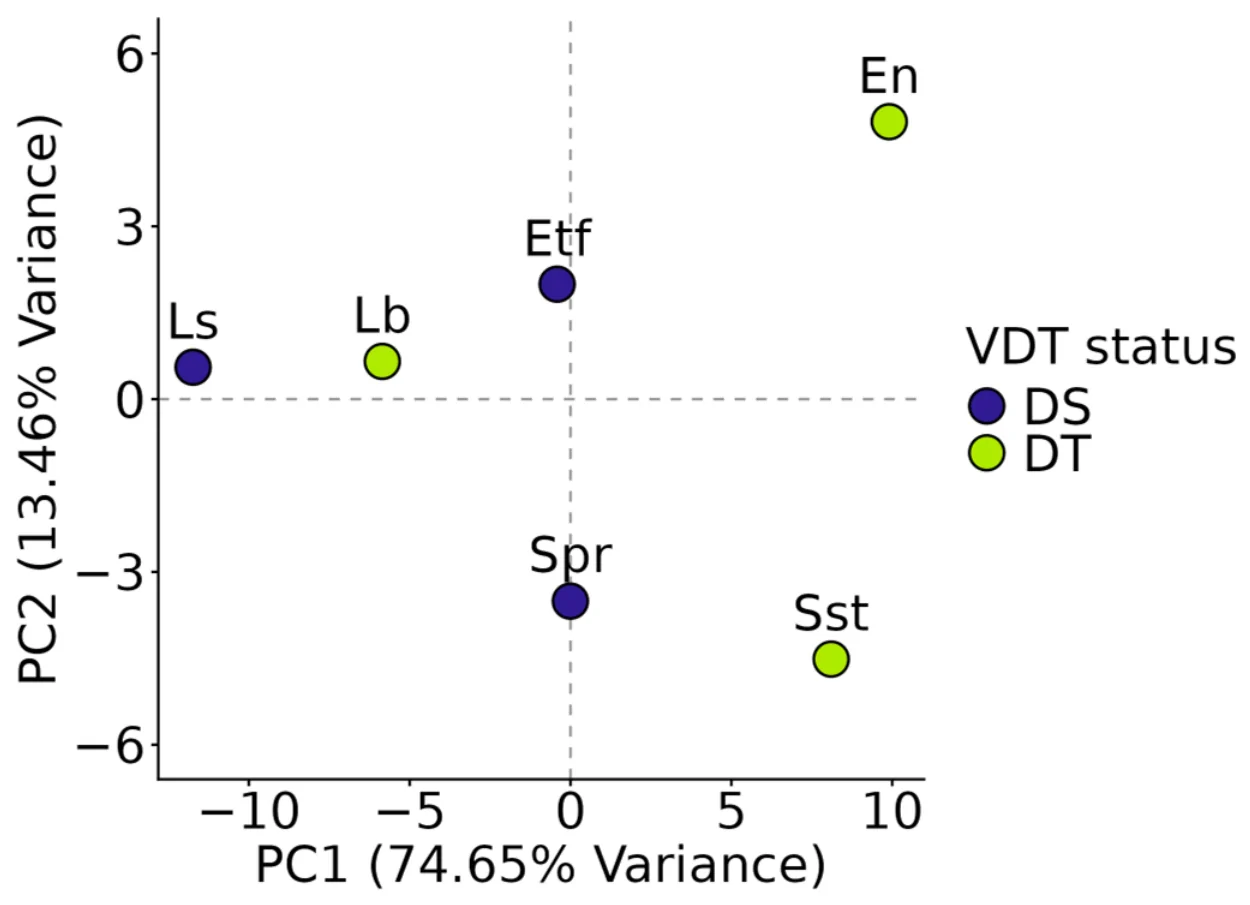
Principal component analysis (PCA) of the six species from the three DT/DS sister pairs (*L. brevidens*/*L. subracemosa*, *E. nindensis*/*E. tef*, and *S. stapfianus*/*S. pyramidalis*), based on paralog copy numbers within conservatively regulated orthogroups with higher DT representation. Dashed lines indicate the zero value on each principal component axis.

Hierarchical clustering of the same orthogroup subset (Figure 3) strongly corroborates the PCA findings.

Species cluster strictly according to taxonomic lineage: the two DT grass representatives (En and Sst) form a distinct cluster characterized by predominantly elevated copy numbers; their corresponding DS counterparts (Etf and Spr) form an adjacent group with intermediate values; and Lb and Ls comprise a separate eudicot cluster, albeit with internal contrast (Lb exhibiting generally higher values than Ls). The monocot clade (En/Sst and Etf/Spr) merges prior to joining the eudicot lineage (Lb/Ls), consistent with copy-number profiles tracking taxonomic lineage (the monocot/eudicot split) more closely than convergent adaptation for desiccation tolerance in this dataset.

**Figure 3:**
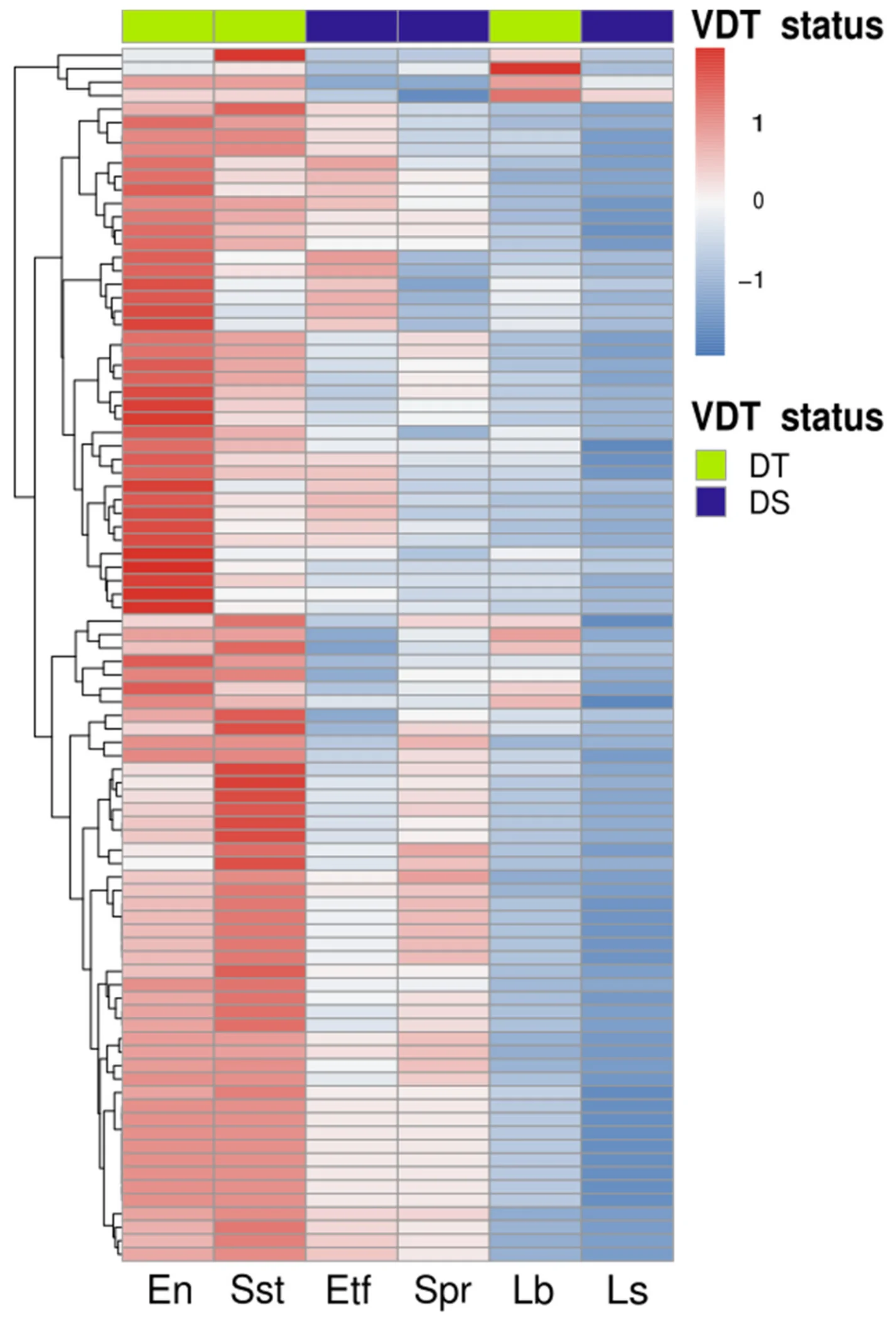
Hierarchical clustering of conservatively regulated orthogroups across the three DT/DS sister-pair species based on paralog representation. The colored bar above the column dendrogram indicates each species’ DT/DS status (Status: DT, lime green; DS, deep purple).

### 3.4. Mapping to Canonical Desiccation-Related Gene Families

To contextualize the observed orthogroup representation profiles (Section 3.3) within the established literature on desiccation tolerance, we compiled a consensus set of eleven canonical gene families and functional protein classes (Table 2). These families encompass key physiological pathways involved in desiccation tolerance, including photoprotection, macromolecular stabilization, seed-developmental co-option, multi-stress signaling, cellular recycling, and redox homeostasis. Table 3 delineates their primary biological functions alongside key supporting literature, serving as a conceptual framework for the quantitative paralog dosage assessment in Section 3.5.

**Table 2:** Canonical gene families and functional protein classes associated with vegetative desiccation tolerance.

Key Reference	Primary Biological Processes and Mechanism	Gene Family
[8,15,16,37,38]	Photoprotection: bind free chlorophyll and stabilize photosynthetic complexes; prevent photo-oxidative damage during desiccation.	ELIP (Early Light-Induced Proteins)
[8,13,15,37]	Molecular chaperones: stabilize membranes and proteins; act as “water replacements” preventing molecular aggregation.	LEA (Late Embryogenesis Abundant proteins)
[8,13,15,37]	Co-option of seed programs: master regulators rerouting seed protective mechanisms into vegetative tissue.	ABI3 and ABI5 (transcription factors)
[15]	Multi-stress signaling: central hub for desiccation, cold and darkness signaling; participates in ABA-dependent responses.	PP2C (Protein Phosphatase 2C)
[13,15,37]	Cellular recycling: manage cellular starvation, recycle damaged components, suppress senescence.	ATG (autophagy-related genes—ATG5, ATG8)
[9]	Protein synthesis: provide a surplus of ribosomes for rapid recovery of protein synthesis upon rehydration.	5S rRNA genes
[8,37]	Lipid body stabilization: protect membrane integrity by coating oil bodies.	Oleosins and caleosins
[39]	Sugar metabolism: facilitate accumulation of octulose, a metabolic reservoir for sucrose.	Transketolases (TKT7, TKT10)
[37,40]	Redox homeostasis: detoxification of reactive oxygen species; often constitutively high expression.	ROS network (SOD, catalases, peroxidases)
[15]	Regulatory adaptation: tolerance to high light intensity and large-scale transcriptional reprogramming.	PP7L, WRKY, FRS

### 3.5. Trend-Conforming Paralog Dosage in Canonical Orthogroups

A one-sided exact Mann–Whitney test (Wilcoxon rank-sum, alternative = “greater”; Table 3) compares, across 32 canonical orthogroups, the mean number of genes per species whose individual regulatory direction matches the orthogroup’s dominant trend (trend-conforming paralogs, see definition in Section 2.8 and Section 4.1), between DT (n = 9 species) and DS (n = 3 species). At an uncorrected threshold of *p* < 0.05, using a genuinely exact one-sided test, three orthogroups show the strongest directional (nominal) support for higher dosage in DT: dehydrin (OG0001406; DT mean 2.33 vs. DS 0.00; *p* = 0.0182), a NAC-domain-containing protein (OG0000837; DT mean 2.44 vs. DS 0.00; *p* = 0.0455) and a LEA hydroxyproline-rich glycoprotein (OG0002251; DT mean 1.22 vs. DS 0.00; *p* = 0.0455). Two orthogroups reported as nominal in an earlier, normal-approximation-based version of this test (ascorbate peroxidase OG0004304 and LEA-related OG0003891) no longer reach *p* < 0.05 under the exact test (both *p* = 0.0909); see Section 4.1 for the source of this correction. These *p*-values are uncorrected; after Benjamini–Hochberg (BH/FDR) correction for multiple testing, no orthogroup remains significant (the lowest adjusted value is *p*adj ≈ 0.45), so the results here and below are consistently interpreted as directional trends rather than statistically proven differences (the full analysis and mathematical explanation are given in Section 4.3.3). The remaining 29 of 32 tested orthogroups do not reach this uncorrected threshold; most (28 of 29) nonetheless show a directionally higher, non-significant DT mean, and only one orthogroup shows an exactly tied DT/DS mean.

**Table 3:** Exact one-sided Mann–Whitney test for trend-conforming paralog dosage (DT versus DS), with FDR-adjusted *p*-values.

Concordant Species	Trend	*p*adj (FDR)	*p*	W	DS Mean	DT Mean	Gene Family	Orthogroup
8	UU	0.450	0.0182	25.5	0.00	2.33	Dehydrin family protein	OG0001406
7	ND	0.450	0.0455	24.0	0.00	2.44	NAC domain containing protein	OG0000837
7	ND	0.450	0.0455	24.0	0.00	1.22	LEA hydroxyproline-rich glycoprotein family	OG0002251
7	DD	0.450	0.0727	22.5	0.33	2.22	WRKY DNA-binding protein	OG0000913
6	DD	0.450	0.0909	22.5	0.00	1.11	LEA protein group	OG0003891
6	ND	0.450	0.0909	22.5	0.00	1.11	ascorbate peroxidase	OG0004304
8	UU	0.450	0.1	21.5	0.67	2.44	Late Embryogenesis Abundant 4–5	OG0002477
5	ND	0.455	0.1591	21.0	0.00	0.56	Peroxidase superfamily protein	OG0007823
5	ND	0.455	0.1591	21.0	0.00	0.89	LEA hydroxyproline-rich glycoprotein family	OG0012098
5	UU	0.455	0.1591	21.0	0.00	0.78	LEA family protein	OG0010939

W, Mann–Whitney *U* test statistic; *p*-value, exact one-sided uncorrected *p*-value; *p*_adj_, FDR-adjusted *p*-value (Benjamini–Hochberg). Regulatory trend: Two-letter code denoting directional transcriptional response under dehydration and desiccation states: UU, upregulated–upregulated; DD, downregulated–downregulated; ND, non-significant–downregulated (non-significantly expressed under dehydration and downregulated under desiccation); NU, non-significant–upregulated (non-significantly expressed under dehydration and upregulated under desiccation); Section 2.4. Concordant species: number of species displaying paralogs matching the dominant orthogroup trend. Shown are the ten canonical orthogroups with the lowest adjusted *p*-values (of 32 canonical orthogroups tested; see Supplementary Table S5 for complete results).

Notably, the three orthogroups showing nominal support under the corrected exact test span distinct functional categories—structural protection (dehydrin, LEA hydroxyproline-rich glycoprotein) and transcriptional control (NAC)—indicating that paralog enrichment, where present, is not restricted to a single protective pathway (Section 4.2). All three are supported at the individual species level by a majority of DT species (dehydrin OG0001406: 8 of 9; NAC-domain OG0000837 and LEA OG0002251: 7 of 9 each), and two of the three (OG0000837, OG0002251) are additionally concordant across all three matched DT/DS sister pairs (Section 4.1), while OG0001406 is concordant in two of the three pairs. Orthogroups supported by fewer species or fewer matched pairs require correspondingly more cautious interpretation (Section 4.3.3). As these orthogroups were preselected for higher DT than DS representation (Section 2.7), these results are best interpreted as a hypothesis-generating exploration of that pattern rather than an independent statistical confirmation of DT-associated enrichment (Section 2.8).

## 4. Discussion

### 4.1. Methodological Considerations: Integrating Genome Assemblies and De Novo Transcriptomes

The quantitative measure used in Section 3.3 and Section 3.5 for “higher paralog representation” is not a purely structural genomic metric but derives directly from the classification pipeline described in Section 2.4 and Supplementary Methods S1: genes are classified by regulatory direction under dehydration and desiccation, grouped into orthogroups, and a dominant regulatory trend is assigned to each orthogroup. The “paralogs” counted in Table 2 and Table 3 therefore represent genes that (i) are present in the species’ available genome or transcriptome and (ii) are transcriptomically classified as following the dominant regulatory trend of the corresponding orthogroup, not a simple structural copy count. We therefore replace the term “active paralogs” throughout with the more precise and unambiguous trend-conforming paralogs (paralogs following the orthogroup’s dominant regulatory trend). An additional consequence of this design is that the reference trend used to classify ‘trend-conforming’ status is derived exclusively from the DT species (Section 2.4); a lower trend-conforming paralog count in DS species may therefore partly reflect a genuinely different, or more limited, transcriptional response to advanced desiccation stress in DS species themselves, rather than solely a smaller underlying paralog repertoire. This limitation should be kept in mind when interpreting DT–DS differences in Table 3. This hybrid, rather than purely genomic, nature of the metric reinforces the significance of the methodological heterogeneity in Table 1: for the four transcriptome-only species (Cr, Lb, and Ls, with genome sequences available but without a reliable gene annotation (see Section 2.2), and Xh, for which no genome sequence has been published), the number of trend-conforming paralogs depends entirely on the completeness of the *de novo* assembled transcriptome; gene copies not expressed at the time of sampling cannot be captured either structurally or transcriptomically, and their repertoire is likely underestimated. Because the sister pair Lb/Ls is entirely transcriptome based while En/Etf and Sst/Spr are entirely genome based, the DT/DS comparison in Section 3.5 is not methodologically symmetric across the three sister pairs, a limitation that must be stated explicitly when interpreting the Mann–Whitney results. The same three transcriptome-only taxa (*Cr. plantagineum*, *L. brevidens*, *L. subracemosa*) also show reduced phylogenetic resolution among themselves in the branch-support analysis (Section 3.1; Supplementary Table S3), consistent with *de novo* assemblies carrying less complete or less reliable orthology signal than genome-based gene models, though a genuinely short divergence interval between these lineages cannot be excluded as an alternative (non-methodological) explanation for the same weak resolution. A separate methodological feature is that the Mann–Whitney test is one-sided (alternative = “greater”), testing specifically the hypothesis that DT species carry more trend-conforming paralogs than DS species, rather than a two-sided difference, and precluding detection of an effect in the opposite direction (DS > DT). A substantial methodological limitation follows directly from the size of the DS group (*n* = 3): for the exact one-sided Mann–Whitney test with *n*_DT = 9 and *n*_DS = 3, the smallest theoretically achievable *p*-value is

$$p_{\min}=\frac{1}{\left(\begin{array}{c} 12\\ 3 \end{array}\right)}=1/220\approx 0.00455,$$

even under complete separation between groups. Because Benjamini–Hochberg correction across 36 simultaneous tests requires the leading result to reach *p* ≤ 0.0014 to survive, it is mathematically impossible for any orthogroup in Table 3 to reach FDR significance under this design, regardless of the true biological effect (the full derivation is given in Section 4.3.3). Mann–Whitney results are therefore interpreted throughout the paper as directional (nominal) trends rather than statistically proven differences.

The primary comparison in Section 3.5 retains all nine DT and three DS species, including those represented only by *de novo* transcriptomes, for two reasons. First, restricting the analysis to genome-annotated taxa alone would discard four of the twelve species examined—Cr, Lb, and Xh among the DT group and Ls among the DS group—removing over a third of the taxonomic and phylogenetic breadth that motivates this comparative study (Section 1). Second, it would shrink an already small DS group (*n* = 3) to *n* = 2, reducing rather than improving statistical power. We therefore report the full nine-versus-three comparison as the primary analysis and additionally repeated it restricted to the six DT and two DS species with genome-based gene models (Bh, En, Hrh, Oth, Sst, Xs versus Etf, Spr), as a robustness check.

This stricter subset changes which orthogroups reach nominal significance rather than uniformly confirming or overturning the full-sample result: dehydrin (OG0001406) and LEA4-5 (OG0002477) remain nominal (*p* = 0.036) the combinatorial minimum for this *n* = 6 vs. *n* = 2 design,

$$p_{\min}=\frac{1}{\left(\begin{array}{c} 8\\ 2 \end{array}\right)}=1/28\approx 0.03571,$$

while NAC-domain OG0000837 and LEA OG0002251 do not (*p* ≈ 0.11)—plausibly because Ls, being transcriptome-only, may itself underestimate DS-side paralog counts for some orthogroups (Section 2.2), partly inflating the apparent DT-DS contrast for those loci in the full sample. Critically, no orthogroup approaches FDR significance in either version of the test (genome-only *p*_min = 1/28 ≈ 0.036 is still far above the ≈0.0014 threshold required to survive Benjamini–Hochberg correction across 36 tests), so the qualitative conclusion—a directional, not statistically proven, signal—is robust to this restriction, even though the specific leading candidates shift somewhat with sample composition.

### 4.2. Functional Regulation Versus Structural Paralog Capacity in Desiccation Tolerance

Because the metric in Section 3.5 already embeds the regulatory classification from the differential expression analysis (Section 2.4, Supplementary Methods S1), the Mann–Whitney test does not compare two fully independent lines of evidence but rather tests a narrower question: whether DT species carry more gene copies following the orthogroup’s consensus regulatory trend than DS species. Three of 32 canonical orthogroups show the strongest directional (nominal) support in this direction under the corrected exact test (all DT > DS), though none survives correction for multiple testing (see Section 4.1 and Section 4.3.3); given a design with *n* = 3 DS species this is expected, and not necessarily a sign of the lack of a real effect. With this caveat, the nominal picture is nevertheless consistent with the notion that the established regulatory conservation is, in part, accompanied by a greater number of corresponding copies in tolerant species; for the majority (29 of 32), however, conserved regulation is not accompanied even nominally by higher trend-conforming paralog dosage relative to DS species, i.e., regulatory conservation does not necessarily require a larger gene repertoire. The case of OG0000837 (a NAC-domain transcription factor, trend ND: not differentially expressed during dehydration, but downregulated during desiccation) is particularly instructive: despite this loss of expression as desiccation deepens, the number of trend-conforming paralogs is nominally higher in DT species (*p* = 0.0455; concordant across all three matched sister pairs, Section 4.1), one of only three canonical orthogroups retaining nominal support under the corrected exact test (Section 4.3.3). An expanded repertoire that is progressively silenced as desiccation progresses is not an intuitive result; a plausible (though at this stage hypothetical) explanation is that a larger copy number provides regulatory reserve or buffering capacity, allowing fine control over which paralogs remain active as water content falls, rather than directly driving stronger induction, a hypothesis broadly consistent with gene-dosage/balance theory for gene duplication [41]. NAC transcription factors are established multifunctional regulators of both positive and negative stress responses in plants, lending biological plausibility to a duplication-enabled repertoire from which desiccation-specific repression, rather than induction, may be selected. A related, though weaker, pattern is seen in OG0003891 (a LEA-related orthogroup, trend DD: suppressed at both stress stages): its trend-conforming paralog count remains descriptively higher in DT than DS species (DT mean 1.11 vs. DS mean 0.00), though it no longer reaches nominal significance under the corrected exact test (*p* = 0.0909; Section 4.3.3).

### 4.3. Distinct Mechanisms Across Canonical Desiccation-Tolerance Families

#### 4.3.1. OG0000271 (ELIP1/ELIP2): An Expansion Characteristic of the Grasses (Poaceae), Not Strictly DT-Specific

OG0000271 contains exactly two *A. thaliana* genes, ELIP1 and ELIP2, and therefore corresponds directly to the ELIP (Early Light-Induced Proteins) orthogroup itself, rather than to the broader LHC/CAB superfamily. The gene counts in this orthogroup can accordingly be read as a direct measure of ELIP copy number in each species, without requiring further filtering. Set against the chlorophyll strategy in Table 1 (HDT versus PDT) and taxonomic affiliation, OG0000271 values trace a clear pattern confined to the Chloridoideae grasses: En (DT, PDT) = 28, Sst (DT, HDT) = 28, Spr (DS) = 24, Etf (DS) = 9, that is, only one of the two DS species (Spr) approaches the values of the DT grasses, while the other (Etf) remains close to the levels observed outside Poaceae. Outside Poaceae, the pattern does not carry over: the eudicots Lb (DT) = 6 and Ls (DS) = 5 remain low and similar to one another, and the two Xerophyta species (DT, PDT, but not Poaceae) likewise remain low: Xh = 7, Xs = 9. In other words, expansion of ELIP1/ELIP2 correlates more closely with taxonomic membership in the grasses (Poaceae) than with desiccation tolerance or homoiochlorophyllous/poikilochlorophyllous status. This result contrasts with the broader genome-wide finding of VanBuren [6] that ELIP genes are expanded across all surveyed resurrection plants relative to their sensitive relatives (10–26 versus 1–8 copies): in the present sample, this pattern is not uniformly confirmed, most likely because the sample here includes only three DS species (one of which, Spr, is itself a grass), whereas VanBuren [6] covers a broader and phylogenetically more diverse set of sensitive comparator species.

#### 4.3.2. Conserved Expression Trends Across Core Protective Families (LEA, Dehydrins, ROS Scavengers): Integrating Transcriptomic and Genomic Perspectives

The present paper combines the transcriptomic analysis of regulatory conservation (Section 2.4, Supplementary Methods S1) with a genomic analysis of the structural capacity of the corresponding gene families. Of the eleven LEA/dehydrin orthogroups conservatively regulated according to Supplementary Table S6, only two (dehydrin OG0001406 and the LEA-related OG0002251) show a nominally higher number of trend-conforming paralogs in DT species under the corrected exact test (uncorrected *p* < 0.05; Table 3), neither of which survives FDR correction (Section 4.3.3); the antioxidant system representative considered here, ascorbate peroxidase (OG0004304), was nominal under an earlier, normal-approximation-based version of the test but no longer reaches *p* < 0.05 under the exact test (*p* = 0.0909; Section 4.1). The remaining members of these families retain the established conserved regulation without this being accompanied even nominally by higher trend-conforming paralog dosage relative to DS species. This partial, rather than complete, agreement between the two lines of evidence supports the conclusion of Section 4.2: conservation of the regulatory response and expansion of the gene repertoire are related but not necessarily jointly present for every individual member of a given family.

#### 4.3.3. Directional Trends in Trend-Conforming Paralog Dosage in a Subset of Canonical Orthogroups (Mann–Whitney Test, with FDR Correction)

The quantitative evaluation relies on an exact, one-sided Mann–Whitney U test applied to a preselected set of canonical orthogroups rather than a genome-wide scan. While the analytical pipeline was executed genome-wide across all 21,517 orthogroups, Table 3 specifically reports the candidate subset with direct literature relevance (32 canonical orthogroups evaluated out of 36 in the source pipeline; the four additional loci (OG0001634, OG0009366, OG0000423, and OG0002497) were omitted from Table 3 because they fall outside the core conservatively regulated set and represent candidates for future study). The analytical framework originated from the three DT/DS sister pairs (Lb/Ls, Sst/Spr, En/Etf; six species) and was subsequently expanded to the full nine DT versus three DS comparative framework to evaluate gene repertoire expansion over a broader phylogenetic sample.

At uncorrected thresholds, three of the 32 tested orthogroups reach *p* < 0.05 under the corrected exact test, all displaying a positive directional trend (DT > DS). Following Benjamini–Hochberg (BH/FDR) correction within the targeted set of 36 hypothesis tests, the lowest adjusted value is padj ≈ 0.45 (shared by eight of the 32 canonical orthogroups (all those with uncorrected *p* ≤ 0.10), including dehydrin OG0001406). Corrected across the full genome-wide set of 21,517 tests, the adjusted values are, unsurprisingly, even further from significance, underscoring that genome-wide screening does not reveal a stronger signal than the targeted canonical analysis. Consequently, no individual orthogroup achieves statistically significant separation at *p*adj < 0.05 after controlling for multiple testing.

As detailed in Section 4.1, the current design (nDS = 3) prevents definitive FDR-controlled inference; low statistical power explains why a true effect may fail to reach significance, but it does not itself provide evidence that the underlying biological effect is strong, weak, or even present. Accordingly, the quantitative findings in Table 3 are presented strictly as directional nominal trends. The three top-ranked orthogroups with uncorrected *p* < 0.05 under the corrected exact test (dehydrin OG0001406, NAC transcription factor OG0000837, and LEA hydroxyproline-rich glycoprotein OG0002251) represent candidate loci with the strongest directional support for elevated trend-conforming paralog dosage in desiccation-tolerant species, serving a hypothesis-generating role for future functional validation. This framing is consistent with, and directly informed by, independent evidence from the broader gene family-evolution literature: comparative genomic analyses across 60 plant genomes have documented genomic diversification and duplication-driven expansion of the Dehydrin and LEA_2 subfamilies as a general feature linked to desiccation/abiotic-stress adaptation [42]; NAC transcription factors were previously shown to be induced as part of the dehydration-responsive transcriptional reprogramming of *H. rhodopensis*, one of the nine DT species analyzed here [43]; and functional validation of tandemly duplicated dehydrin paralogs by overexpression and gene silencing in an unrelated drought-tolerant shrub has directly confirmed the type of dosage-dependent phenotype predicted by the present analysis [44].

### 4.4. Limitations

The primary limitations of this study encompass four key methodological and structural aspects:

- Input data heterogeneity: Three DT species (Cr, Lb, and Xh) and one DS species (Ls) rely on *de novo* transcriptome assemblies rather than structural genome annotations, which likely leads to an underestimation of their true gene copy repertoires (Section 4.1). For Cr, Lb, and Ls, this reflects the current absence of reliable, publicly available gene annotations for their respective genome assemblies (Section 2.2), whereas for Xh, no genome sequence has been published to date.
- Methodological asymmetry across sister pairs: The eudicot Lb/Ls sister pair is entirely transcriptome based, whereas the grass pairs (En/Etf and Sst/Spr) are fully genome annotated, introducing structural asymmetry across the pairwise comparative framework; the same transcriptome-only composition may also contribute to why the exact phylogenetic placement of *L. subracemosa* within the Craterostigma/Lindernia clade is only moderately resolved (Section 3.1, Supplementary Table S3), alongside the alternative possibility of a genuinely short divergence interval between these lineages.
- Small desiccation-sensitive sample size: The limited DS cohort size (*n* = 3 species) constrains the statistical power of the Mann–Whitney U test, rendering FDR significance mathematically unattainable under multiple testing conditions across dozens of simultaneous orthogroup comparisons (Section 4.1 and Section 4.3.3).
- Orthogroup annotation strategy: Gene family annotations were assigned via a single *A. thaliana* ortholog (Section 2.5), which in specific instances may cluster functionally heterogeneous paralogs within broader gene families. Consequently, functional conclusions for individual orthogroups should be interpreted as hypothesis-generating insights for future targeted experimental validation rather than definitive proof.

## 5. Conclusions

The nine desiccation-tolerant (DT) species evaluated in this study provide a heterogeneous genomic and transcriptomic dataset against which conservatively regulated orthogroups can be assessed for trend-conforming paralog dosage. Nominal directional support for higher paralog dosage, using a genuinely exact statistical test, is restricted to three canonical orthogroups: a dehydrin, a NAC domain transcription factor, and a LEA hydroxyproline-rich glycoprotein. Due to the inherent sample design constraint (n = 3 DS species) and the resulting combinatorial floor on the exact test’s minimum achievable *p*-value, these nominal trends cannot achieve significance following multiple-testing correction, and are thus framed as hypothesis-generating insights rather than statistically proven differences.

Importantly, the majority of conservatively regulated orthogroups maintain highly conserved transcriptional responses under dehydration and desiccation without exhibiting higher observed paralog representation, including OG0000271 (ELIP1/ELIP2), whose expansion primarily reflects lineage-specific evolution within Poaceae rather than desiccation tolerance per se.

Collectively, these findings provide an integrated transcriptomic and genomic evaluation of paralog representation across protective gene families, establishing a prioritized set of candidate loci to guide future functional studies and broader phylogenetic comparisons.

## Data Availability

The genome assemblies and annotations used in this study are publicly available under the BioProject accessions listed in the corresponding cited publications (Table 1); for *Cr. plantagineum*, *L. brevidens* and *L. subracemosa*, only the underlying genome sequence assemblies (not gene annotations) are available under these accessions, and gene-level data for these species derive instead from the *de novo* assembled transcriptomes described in Section 2.2 and Supplementary Methods S1. Most conservatively regulated gene families during desiccation listed in Supplementary Table S4. Conserved regulated orthogroups, where genes in tolerant species are more abundant than those in desiccation-sensitive orthogroups, are listed in Supplementary Table S6. The raw RNA-seq data used for the differential expression analysis (Section 2.4, Supplementary Methods S1) are publicly available in NCBI SRA under the BioProject accessions listed in Supplementary Table S2. The analysis code is not yet in a public repository; it will be made available upon reasonable request to the corresponding author, and a public repository is in preparation for release upon publication.

## Supplementary Materials

The following supporting information can be downloaded at https://www.mdpi.com/article/10.3390/genes17101202/s1, Supplementary Methods S1: Differential expression analysis, orthogroup analysis, and consolidation of regulatory trends under dehydration and desiccation (full protocol for Section 2.4 and Section 2.5) [13,19,20,26,45,46,47,48,49,50,51,52,53,54,55,56,57,58,59,60,61,62,63,64,65,66,67]; Supplementary Table S1: Number of RWC sampling time points per desiccation-tolerant species; Supplementary Table S2: BioProject accessions for the transcriptomic (RNA-seq) datasets used in the differential expression analysis [8,9,12,13,14,68,69,70,71]; Supplementary Table S3: Branch (node) support values for the species tree in Figure 1, from IQ-TREE 2 ultrafast bootstrap analysis; Supplementary Table S4: Most conservatively regulated gene families during desiccation; Supplementary Table S5: Complete exact one-sided Mann–Whitney test results for trend-conforming paralog dosage (DT versus DS) across all 32 canonical orthogroups; Supplementary Table S6: Paralog (gene copy) counts per orthogroup in matched desiccation-tolerant/desiccation-sensitive species pairs, relative to eudicot and monocot model genomes.

## Abbreviations

The following abbreviations are used in this manuscript:

VDT	Vegetative desiccation tolerance
DT	Desiccation-tolerant
DS	Desiccation-sensitive
RWC	Relative water content
DE	Differential expression
FDR	False discovery rate
BH	Benjamini–Hochberg (procedure)
PCA	Principal component analysis
TF	Transcription factor
OG	Orthogroup
HDT	Homoiochlorophyllous desiccation tolerance
PDT	Poikilochlorophyllous desiccation tolerance
LEA	Late Embryogenesis Abundant (protein)
ELIP	Early Light-Induced Protein
GO	Gene Ontology
