# Supplementary material for: Genomic and Transcriptomic Analysis of Nine Resurrection Plant Species: Conserved Regulation Versus Structural Paralog Capacity in Desiccation Tolerance
Source: Genes (Basel). 2026 Sep 29;17(10):1202. doi: 10.3390/genes17101202 (PMC13704565; doi:10.3390/genes17101202)
Supplement: Supplementary file 1 [file genes-17-01202-s001.zip › Supplementary Methods S1.pdf]

Supplementary Methods S1

Differential expression analysis, orthogroup analysis, and consolidation of regulatory trends under dehydration and desiccation

This document describes in full the bioinformatic pipeline used to identify orthogroups with a conservatively regulated response to dehydration and desiccation across the nine desiccation-tolerant (DT) species examined, summarised briefly in the main text of the paper (2.4-2.5). The protocol was developed and applied by the authors of the present study as part of a broader research programme on the regulatory mechanisms of desiccation tolerance, and is an integral methodological element of the present paper.

S1.1. Samples and stress stages

Samples from drought experiments were used, spanning a well-hydrated condition and progressively decreasing RWC to an air-dry condition. For the purposes of the analysis, samples were grouped into two stress stages relative to the hydrated baseline: dehydration (RWC > 40%) and desiccation (RWC < 40%). The 40% RWC threshold reflects the physiological transition at which most of the species examined move from active photosynthetic activity to the metabolically quiescent state characteristic of the desiccation phase [13, 45, 46, 47, 48].

S1.2. Data acquisition, quality control and trimming

Raw sequencing reads for each species were downloaded from the NCBI SRA database using SRA Toolkit v2.11.3 [49]. The corresponding BioProject accessions for each species are listed in Supplementary Table S3. Quality control and removal of adapter and low-quality sequence were performed with Trimmomatic v0.39 [50].

S1.3. *De novo* transcriptome assembly

For the three DT species without a published genome annotation (*Craterostigma plantagineum*, *Lindernia brevidens*, *Xerophyta humilis*) and for the DS species *Lindernia subracemosa*, transcriptomes were assembled *de novo* with Trinity v.2.10.0 [51], using default parameters (see also 2.2 of the main text).

S1.4. Functional annotation of the *de novo* assembled transcriptomes

Predicted transcripts from the *de novo* assembled transcriptomes were annotated by: identification of coding regions with TransDecoder v5.5.0 [52]; homology searches against the NCBI nr [53] and UniProt [54] databases using DIAMOND v2.1.21[55]; protein domain searches against the Pfam 37.0 database [56] using HMMER v3.3.2 [57];

signal peptide prediction with SignalP 6.0 [58]; and transmembrane domain prediction with TMHMM v2.0 [59,60].

S1.5. Transcriptome completeness assessment

The completeness of each *de novo* assembled transcriptome was assessed with BUSCO v5.6 [61] against the embryophyta_odb10 lineage dataset of conserved single-copy orthologous genes [61].

S1.6. Orthogroup analysis

Genes from all 20 angiosperm species examined (nine DT, three DS, and eight additional species providing broader phylogenetic context - see 2.1 of the main text) were grouped into orthogroups using OrthoFinder v2.5.4 [19], based on protein sequences from the available genome annotations or open reading frames (ORFs) predicted from the *de novo* transcriptomes. The species tree derived from the same analysis was rooted on the basal angiosperm *Amborella trichopoda* and visualised in iTOL [20] Figure 1 of the main text.

S1.7. Transcript quantification and gene-level summarisation

Transcript abundance was quantified with Salmon v1.10 [62]. Transcript-to-gene summarization was performed with tximport v1.38.2 [63] based on transcript-to-gene mapping (tx2gene) generated by RapClust v0.1.2 [67] for *de novo* assembled transcriptomes or with GFFread v0.12.2 [64] for species with sequenced genomes.

S1.8. Differential expression analysis

Differential expression was determined using the RNAseqV2 pipeline [65], with quantification data imported via tximport and statistical testing performed with DESeq2 v1.50.2 [66]. For each gene, differential expression relative to the well-hydrated baseline was determined separately for each sample. Based on the magnitude and direction of gene expression change, genes were classified into three categories: up (U, $\log_2 FC > 1$), down (D, $\log_2 FC < -1$), and unchanged (N, $-1 < \log_2 FC < 1$), at a statistical significance threshold of $p_{adj} < 0.05$ (corrected for multiple testing in each species comparison). For the species involved in the study, different numbers of samples were available for a given phase of drought. For each gene and stage, two parallel trend profiles were constructed - max (based on the highest observed $\log_2 FC$ value of dehydration and desiccation) and min (the lowest) - to capture the variations between samples, rather than randomly choosing one representative result. The three values of differential gene expression and the two stages in which we examine gene expression generate 9 different patterns of gene expression regulation, which in our study are denoted by two-letter abbreviations (DD - gene down-regulated in the dehydration and desiccation stage; ND - gene not

differentially expressed during dehydration, but down-regulated during desiccation; NU - gene not differentially expressed during dehydration, but up-regulated during desiccation, etc.). Genes following the NN model (not differentially expressed at both stages) were omitted from the study, as the analysis only included differentially expressed genes. We formed a consolidated database in which gene expressions for up-regulated trends (UU, NU, UN and DU) were extracted from the max and down-regulated ones (UU, NU, UN and UD).

S1.9. Consolidation of trends at orthogroup level and definition of conservatively regulated orthogroups

For each orthogroup containing at least one differentially expressed gene in at least one of the nine DT species, a dominant trend was defined as the mode (most frequent value) among the eight possible trend codes across the individual genes of the orthogroup in the nine DT species. For each orthogroup, a conservation criterion (Score) was calculated as the mean percentage of genes per species (averaged across the nine DT species) following the orthogroup's dominant trend. A second measure, "Concordant Species", records the number of species (out of a maximum of nine) in which the orthogroup's dominant trend was in fact dominant at the individual species level as well (not only in the pooled sample). A threshold of Score ≥ 35.19 (the 90th percentile of the Score distribution across all orthogroups with at least one DE gene) narrowed the initial set of approximately 2,010 conservative orthogroups.

S1.10. Functional annotation of orthogroups at gene-family level

Functional annotation of orthogroups (see 2.5 of the main text) was carried out through automated queries to the Ensembl Plants database [26] via the Ensembl REST API [26], based on the *Arabidopsis thaliana* genes contained within each orthogroup.

References

13 Costa, M.-C.D.; Artur, M.A.S.; Maia, J.; Jonkheer, E.; Derks, M.F.L.; Nijveen, H.; Williams, B.; Mundree, S.G.; Jiménez-Gómez, J.M.; Hesselink, T.; et al. A Footprint of Desiccation Tolerance in the Genome of *Xerophyta viscosa*. Nat. Plants 2017, 3, 17038, doi:10.1038/nplants.2017.38.

19 Emms, D.M., Kelly, S. OrthoFinder: phylogenetic orthology inference for comparative genomics. Genome Biol 20, 238 (2019). https://doi.org/10.1186/s13059-019-1832-y.

20 Letunic, I.; Bork, P. Interactive Tree of Life (ITOL) v6: recent updates to the phylogenetic tree display and annotation tool. Nucleic Acids Res. 2024, 52, W78–W82, doi:10.1093/nar/gkae268.

26 Yates, A.; Beal, K.; Keenan, S.; McLaren, W.; Pignatelli, M.; Ritchie, G.R.S.; Ruffier, M.; Taylor, K.; Vullo, A.; Flicek, P. The Ensembl REST API: Ensembl Data for Any Language. Bioinformatics 2015, 31, 143–145, doi:10.1093/bioinformatics/btu613.

45 Oliver, M.J.; Guo, L.; Alexander, D.C.; Ryals, J.A.; Wone, B.W.M.; Cushman, J.C. a sister group contrast using untargeted global metabolomic analysis delineates the biochemical regulation underlying desiccation tolerance in *Sporobolus stapfianus*. Plant Cell 2011, 23, 1231–1248, doi:10.1105/tpc.110.082800.

46 Farrant, J.M.; Cooper, K.; Hilgart, A.; Abdalla, K.O.; Bentley, J.; Thomson, J.A.; Dace, H.J.W.; Peton, N.; Mundree, S.G.; Rafudeen, M.S. A molecular physiological review of vegetative desiccation tolerance in the resurrection plant *Xerophyta viscosa* (Baker). Planta 2015, 242, 407–426, doi:10.1007/s00425-015-2320-6.

47 Blomstedt, C.; Griffiths, C.; Gaff, D.; Hamill, J.; Neale, A. Plant Desiccation Tolerance and Its Regulation in the Foliage of Resurrection "Flowering-Plant" Species. Agronomy 2018, 8, 146, doi:10.3390/agronomy8080146.

48 Zhang, Q.; Bartels, D. Molecular responses to dehydration and desiccation in desiccation-tolerant angiosperm plants. J. Exp. Bot. 2018, 69, 3211–3222, doi:10.1093/jxb/erx489.

49 SRA Toolkit Development Team. SRA-Toolkit. Available online: https://hpc.nih.gov/apps/sratoolkit.html (accessed on 2 November 2023).

50 Bolger, A.M.; Lohse, M.; Usadel, B. Trimmomatic: A flexible trimmer for Illumina sequence data. Bioinformatics 2014, 30, 2114–2120, doi:10.1093/bioinformatics/btu170.

51 Haas, B.J.; Papanicolaou, A.; Yassour, M.; Grabherr, M.; Blood, P.D.; Bowden, J.; Couger, M.B.; Eccles, D.; Li, B.; Lieber, M.; et al. De novo transcript sequence reconstruction from rna-seq using the trinity platform for reference generation and analysis. Nat. Protoc. 2013, 8, 1494–1512, doi:10.1038/nprot.2013.084.

52 Haas, B.J.; Papanicolaou, A. TransDecoder, Version 5.4.0. Available online: https://github.com/TransDecoder/TransDecoder (accessed on 11 September 2026).

53 Goldfarb, T.; Kodali, V.K.; Pujar, S.; Brover, V.; Robbertse, B.; Farrell, C.M.; Oh, D.-H.; Astashyn, A.; Ermolaeva, O.; Haddad, D.; et al. NCBI RefSeq: reference sequence standards through 25 years of curation and annotation. Nucleic Acids Res. 2025, 53, D243–D257, doi:10.1093/nar/gkae1038.

54 Bateman, A.; Martin, M.-J.; Orchard, S.; Magrane, M.; Adesina, A.; Ahmad, S.; Bowler-Barnett, E.H.; Bye-A-Jee, H.; Carpentier, D.; Denny, P.; et al. UniProt: The Universal Protein Knowledgebase in 2025. Nucleic Acids Res. 2025, 53, D609–D617, doi:10.1093/nar/gkae1010.

55 Buchfink, B.; Reuter, K.; Drost, H.-G. Sensitive protein alignments at tree-of-life scale using DIAMOND. Nat. Methods 2021, 18, 366–368, doi:10.1038/s41592-021-01101-x.

56 Mistry, J.; Chuguransky, S.; Williams, L.; Qureshi, M.; Salazar, G.A.; Sonnhammer, E.L.L.; Tosatto, S.C.E.; Paladin, L.; Raj, S.; Richardson, L.J.; et al. Pfam: The protein families database in 2021. Nucleic Acids Res. 2021, 49, D412–D419, doi:10.1093/nar/gkaa913.

57 Potter, S.C.; Luciani, A.; Eddy, S.R.; Park, Y.; Lopez, R.; Finn, R.D. HMMER web werver: 2018 Update. Nucleic Acids Res. 2018, 46, W200–W204, doi:10.1093/nar/gky448.

58 Teufel, F.; Almagro Armenteros, J.J.; Johansen, A.R.; Gíslason, M.H.; Pihl, S.I.; Tsirigos, K.D.; Winther, O.; Brunak, S.; von Heijne, G.; Nielsen, H. SignalP 6.0 predicts all five types of signal peptides using protein language models. Nat. Biotechnol. 2022, 40, 1023–1025, doi:10.1038/s41587-021-01156-3.

59 Krogh, A.; Larsson, B.; von Heijne, G.; Sonnhammer, E.L.L. Predicting transmembrane protein topology with a hidden Markov model: application to complete genomes. J. Mol. Biol. 2001, 305, 567–580, doi:10.1006/jmbi.2000.4315.

60 Hallgren, J.; Tsirigos, K.D.; Pedersen, M.D.; Almagro Armenteros, J.J.; Marcatili, P.; Nielsen, H.; Krogh, A.; Winther, O. Deep TMHMM Predicts Alpha and Beta Transmembrane Proteins Using Deep Neural Networks 2022.

61 Manni, M.; Berkeley, M.R.; Seppey, M.; Zdobnov, E.M. BUSCO: Assessing Genomic Data Quality and Beyond. Curr. Protoc. 2021, 1, doi:10.1002/cpz1.323.
62 Patro, R.; Duggal, G.; Love, M.I.; Irizarry, R.A.; Kingsford, C. Salmon provides fast and bias-aware quantification of transcript expression. Nat. Methods 2017, 14, 417–419, doi:10.1038/nmeth.4197.
63 Soneson, C.; Love, M.I.; Robinson, M.D. Differential analyses for RNA-Seq: transcript-level estimates improve gene-level inferences. F1000Res. 2016, 4, 1521, doi:10.12688/f1000research.7563.2.
64 Pertea, G.; Pertea, M. GFF Utilities: GffRead and GffCompare. F1000Res. 2020, 9, 304, doi:10.12688/f1000research.23297.1.
65 Pardo, J.; VanBuren, R. RNAseqV2. Available online: https://github.com/pardojer23/RNAseqV2 (accessed on 11 September 2026).
66 Love, M.I.; Huber, W.; Anders, S. Moderated estimation of fold change and dispersion for RNA-Seq data with DESeq2. Genome Biol. 2014, 15, 1–21.
67 Srivastava, A.; Sarkar, H.; Malik, L.; Patro, R. Accurate, Fast and Lightweight Clustering of de novo Transcriptomes Using Fragment Equivalence Classes. arXiv 2016, arXiv:1604.03250.
