# Supplementary material for: Genomic and Transcriptomic Analysis of Nine Resurrection Plant Species: Conserved Regulation Versus Structural Paralog Capacity in Desiccation Tolerance
Source: Genes (Basel). 2026 Sep 29;17(10):1202. doi: 10.3390/genes17101202 (PMC13704565; doi:10.3390/genes17101202)
Supplement: Supplementary file 1 [file genes-17-01202-s001.zip › Supplementary Table S2.pdf]

Supplementary Table S2. BioProject accessions for the transcriptomic (RNA-seq) datasets used in the differential expression analysis.

Species	Symbol	BioProject	Study	Reference
Dorcoceras hygrometricum (Boea hygrometrica)	Bh	PRJNA210992	Genome-wide transcriptome analysis during dehydration in leaf tissues of *Boea hygrometrica*	[9]
Craterostigma plantagineum	Cr	PRJNA660052	A multi-omics angle sheds light on the molecular mechanisms underlying desiccation tolerance in *Craterostigma plantagineum*	[68]
Eragrostis nindensis	En	PRJNA548129	Transcriptomic study of well-watered and drought-treated *Eragrostis nindensis* plants	[14]
Haberlea rhodopensis	Hrh	PRJNA432419	Transcriptome reprogramming at late stage of desiccation defines the resurrection behavior of *Haberlea rhodopensis*	[69]
Lindernia brevidens	Lb	PRJNA488068	Parallel transcriptomic study of dehydrating and rehydrating drought-sensitive *Lindernia subracemosa* and resurrection *Lindernia brevidens*	[8]
Lindernia subracemosa	Ls	PRJNA488068	Parallel transcriptomic study of dehydrating and rehydrating drought-sensitive *Lindernia subracemosa* and resurrection *Lindernia brevidens*	[8]
Oropetium thomaeum	Oth	PRJNA548527	Transcriptomic study of well-watered and drought-treated *Oropetium thomaeum* plants	[70]
Sporobolus stapfianus	Sst	PRJNA719555	*Sporobolus stapfianus* genome sequencing, assembly, and dehydration/rehydration transcriptomes	[12]
Sporobolus pyramidalis	Spr	PRJNA719804	*Sporobolus pyramidalis* Genome Sequencing and Transcriptome project	[12]
Eragrostis tef	Etf	PRJNA548000	Well-watered and drought treated *Eragrostis tef* (line PI524434) transcriptomic study.	[14]
Xerophyta humilis	Xh	PRJNA505754	Analysis of desiccation tolerance in the embryonic and vegetative tissues of the resurrection plant *Xerophyta humilis*	[71]
Xerophyta schlechteri	Xs	PRJNA706599	Desiccation-driven senescence in the resurrection plant *Xerophyta schlechteri*	[13]

References

8 VanBuren, R.; Man Wai, C.; Pardo, J.; Giarola, V.; Ambrosini, S.; Song, X.; Bartels, D. Desiccation Tolerance Evolved through Gene Duplication and Network Rewiring in Lindernia. Plant Cell 2018, 30, 2943–2958, doi:10.1105/tpc.18.00517.

9 Xiao, L.; Yang, G.; Zhang, L.; Yang, X.; Zhao, S.; Ji, Z.; Zhou, Q.; Hu, M.; Wang, Y.; Chen, M.; et al. The resurrection genome of Boea hygrometrica: A blueprint for survival of dehydration. Proc. Natl. Acad. Sci. U.S.A. 2015, 112, 5833–5837, doi:10.1073/pnas.1505811112.

12 Chávez Montes, R.A.; Haber, A.; Pardo, J.; Powell, R.F.; Divisetty, U.K.; Silva, A.T.; Hernández-Hernández, T.; Silveira, V.; Tang, H.; Lyons, E.; et al. A comparative genomics examination of desiccation tolerance and sensitivity in two sister grass species. Proc. Natl. Acad. Sci. U.S.A. 2022, 119, doi:10.1073/pnas.2118886119.

13 Costa, M.-C.D.; Artur, M.A.S.; Maia, J.; Jonkheer, E.; Derks, M.F.L.; Nijveen, H.; Williams, B.; Mundree, S.G.; Jiménez-Gómez, J.M.; Hesselink, T.; et al. A Footprint of Desiccation Tolerance in the Genome of Xerophyta viscosa. Nat. Plants 2017, 3, 17038, doi:10.1038/nplants.2017.38.

14 Pardo, J.; Wai, C.M.; Chay, H.; Madden, C.F.; Hilhorst, H.W.M.; Farrant, J.M.; VanBuren, R. Intertwined signatures of desiccation and drought tolerance in grasses. Proc. Natl. Acad. Sci. U.S.A. 2020, 117, 10079–10088, doi:10.1073/pnas.2001928117.

68 Xu, X.; Legay, S.; Sergeant, K.; Zorzan, S.; Leclercq, C.C.; Charton, S.; Giarola, V.; Liu, X.; Challabathula, D.; Renaut, J.; et al. Molecular insights into plant desiccation tolerance: transcriptomics, proteomics and targeted metabolite profiling in Craterostigma plantagineum. Plant J. 2021, 107, 377–398, doi:10.1111/tpj.15294.

69 Liu, J.; Moyankova, D.; Lin, C.-T.; Mladenov, P.; Sun, R.-Z.; Djilianov, D.; Deng, X. Transcriptome Reprogramming during severe dehydration contributes to physiological and metabolic changes in the resurrection plant Haberlea rhodopensis. BMC Plant Biol. 2018, 18, 351, doi:10.1186/s12870-018-1566-0.

70 VanBuren, R.; Wai, C.M.; Zhang, Q.; Song, X.; Edger, P.P.; Bryant, D.; Michael, T.P.; Mockler, T.C.; Bartels, D. Seed desiccation mechanisms co-opted for vegetative desiccation in the resurrection grass Oropetium thomaeum. Plant Cell Environ. 2017, 40, 2292–2306, doi:10.1111/pce.13027.

71 Lyall, R.; Schlebusch, S.A.; Proctor, J.; Prag, M.; Hussey, S.G.; Ingle, R.A.; Illing, N. Vegetative desiccation tolerance in the resurrection plant Xerophyta humilis has not Evolved through reactivation of the seed canonical LAFL regulatory network. Plant J. 2020, 101, 1349–1367, doi:10.1111/tpj.14596.
